# InsuOnline, a Serious Game to Teach Insulin Therapy to Primary Care Physicians: Design of the Game and a Randomized Controlled Trial for Educational Validation

**DOI:** 10.2196/resprot.2431

**Published:** 2013-01-21

**Authors:** Leandro Arthur Diehl, Rodrigo Martins Souza, Juliano Barbosa Alves, Pedro Alejandro Gordan, Roberto Zonato Esteves, Maria Lúcia Silva Germano Jorge, Izabel Cristina Meister Coelho

**Affiliations:** ^1^Departamento de Clínica MédicaCentro de Ciências da Saúde (CCS)Universidade Estadual de Londrina (UEL)Londrina, PRBrazil; ^2^Projeto Pró-Ensino na SaúdePrograma de Pós-Graduação em Saúde da Criança e do AdolescenteFaculdades Pequeno Príncipe (FPP)Curitiba, PRBrazil; ^3^Oniria Software IndustryGames DivisionLondrina, PRBrazil; ^4^Intel Corporation - BrazilSão Paulo, SPBrazil; ^5^Departamento de MedicinaUniversidade Estadual de Maringá (UEM)Maringá, PRBrazil

**Keywords:** Diabetes Mellitus, Insulin, Video Games, Medical Education, Educational Technology, Continuing Medical Education

## Abstract

**Background:**

Physicians´ lack of knowledge contributes to underuse of insulin and poor glycemic control in adults with diabetes mellitus (DM). Traditional continuing medical education have limited efficacy, and new approaches are required.

**Objective:**

We report the design of a trial to assess the educational efficacy of InsuOnline, a game for education of primary care physicians (PCPs). The goal of InsuOnline was to improve appropriate initiation and adjustment of insulin for the treatment of DM. InsuOnline was designed to be educationally adequate, self-motivating, and attractive.

**Methods:**

A multidisciplinary team of endocrinologists, experts in medical education, and programmers, was assembled for the design and development of InsuOnline. Currently, we are conducting usability and playability tests, with PCPs and medical students playing the game on a desktop computer. Adjustments will be made based on these results. An unblinded randomized controlled trial with PCPs who work in the city of Londrina, Brazil, will be conducted to assess the educational validity of InsuOnline on the Web. In this trial, 64 PCPs will play InsuOnline, and 64 PCPs will undergo traditional instructional activities (lecture and group discussion). Knowledge on how to initiate and adjust insulin will be assessed by a Web-based multiple choice questionnaire, and attitudes regarding diabetes/insulin will be assessed by Diabetes Attitude Scale 3 at 3 time points—before, immediately after, and 6 months after the intervention. Subjects´ general impressions on the interventions will be assessed by a questionnaire. Software logs will be reviewed.

**Results:**

To our knowledge, this is the first research with the aim of assessing the educational efficacy of a computer game for teaching PCPs about insulin therapy in DM. We describe the development criteria used for creating InsuOnline. Evaluation of the game using a randomized controlled trial design will be done in future studies.

**Conclusions:**

We demonstrated that the design and development of a game for PCPs education on insulin is possible with a multidisciplinary team. InsuOnline can be an attractive option for large-scale continuous medical education to help improving PCPs´ knowledge on insulin therapy and potentially improving DM patients´ care.

**Trial Registration:**

Clinicaltrials.gov: NCT01759953; http://clinicaltrials.gov/show/NCT01759953 (Archived by WebCite at http://www.webcitation.org/6Dq8Vc7a6).

## Introduction

Achieving and maintaining good glycemic control is the mainstay of treatment of diabetes mellitus (DM) [1]. Unfortunately, only 24-56% of patients with DM are within the goal of the glycated hemoglobin A1c <7%, in most countries [2,3].

Worldwide, according with public health trends, most diabetics are treated by primary care physicians (PCPs) [4], but these professionals lack knowledge and confidence on several aspects of DM management [5], specially regarding insulin use [6]. This contributes to the common problem known as clinical inertia, "the failure to advance therapy when indicated" [7], underuse of insulin [8], and poor glycemic control.

Continuing medical education (CME) on DM and insulin is often advocated as a solution to optimize the knowledge and the practice of PCPs [9]; however, traditional CME activities (such as lectures and group discussions) have small and short-lasting efficacy [10]. Thus, new educational methods are urgently required.

Digital games are currently one of the six greatest trends in higher education [11], since they are able to "create a tight marriage among content, game play, and valued ways of thinking and acting" [12]. One of the reasons for increasing interest in games for higher and professional education is the huge familiarity of most college students with the medium [13]. Most medical students, for instance, even those who do not play video games, have highly favorable views about the use of video games and new technologies in medical education [14]. However, the most compelling reason for adopting learning games, probably, is their pedagogical adequacy. Good learning games are usually built by the same rules that guide the design of effective learning activities, which include stimulus to players´ intrinsic motivation, practice and repetition, effective feedback, arousal of positive feelings, intensity of the experience, and learner choice/involvement [15]. In the medical area particularly, the use of games and simulators for learning clinical skills has the additional advantage of increasing the safety of real patients [16].

In the field of diabetes, some games for education of patients [17-19], and a few technology-based initiatives for education of health professionals [10,19-25] have been described, but to our knowledge, no game have been reported for education of health professionals on diabetes or insulin.

In this paper, we report the design and development of a serious personal computer game for teaching PCPs about initiation and adjustment of insulin in the treatment of DM, and describe the design of a randomized controlled trial to assess if the game can be educationally effective.

## Methods

### Game Design

A multidisciplinary team was assembled, consisting of clinical endocrinologists (LAD, RZE), experts in medical education (PAG, MLSGJ, ICMC) and software developers/game designers (JBA, RMS). The endocrinologists compiled a list of main topics on insulin initiation and adjustment for treatment of adults with DM, in the context of a primary health care setting, outlining the minimum curriculum of the game (Textbox 1).

Periodic team meetings were scheduled during the design and development phases, to review each step, correct problems, and make decisions for the following stages, in an iterative process. The group agreed that the game should be developed in a way to satisfy 2 basic conditions: (1) to be educationally adequate, and (2) to be self-motivating and attractive to the final users.

**The minimum curriculum describing teaching topics on insulin therapy selected for inclusion in the game.**

1. Goals of glycemic control in adults with DM

2. When to start insulin in type 2 DM

3. How to start insulin in type 2 DM (bedtime scheme)

4. Recommendations on insulin use (storing, injection technique, and devices)

5. Hypoglycemia: prevention, recognition, and treatment

6. Self-monitoring of blood glucose

7. Types of insulin: when to use which (NPH, regular)

8. Oral antidiabetic drugs associated with insulin

9. How to adjust insulin dosage

10. How to intensify insulin therapy in type 2 DM (basal-plus scheme)

11. How to start insulin in recent-onset severe type 2 DM (basal-bolus scheme)

12. Type 1 DM: how to recognize it and how to start treatment

13. How to recognize and manage diabetic ketoacidosis

### Storyline

The authors (LAD, RMS) developed the story of InsuOnline based on Joseph Campbell´s classical description of the myth cycle [26]. The story begins in a primary health care clinic, where an experienced medical doctor (the mentor) wants to go on vacation, but he cannot leave without getting a substitute physician to take care of his diabetic patients. So, he randomly chooses a younger doctor to replace him (ie, the player´s avatar). The player proceeds to see a series of diabetic patients who usually require insulin initiation or adjustment in order to obtain a better glycemic control. If the player successfully reaches the end of the game, the mentor character is finally able to go on vacation, and the player´s character is thanked by the mentor, obtains the respect of the nurse and the patients, and a brand new office. The main objective of the game is to have each player correctly initiate or adjust insulin for each patient case.

### Level Design

**Figure 1:**
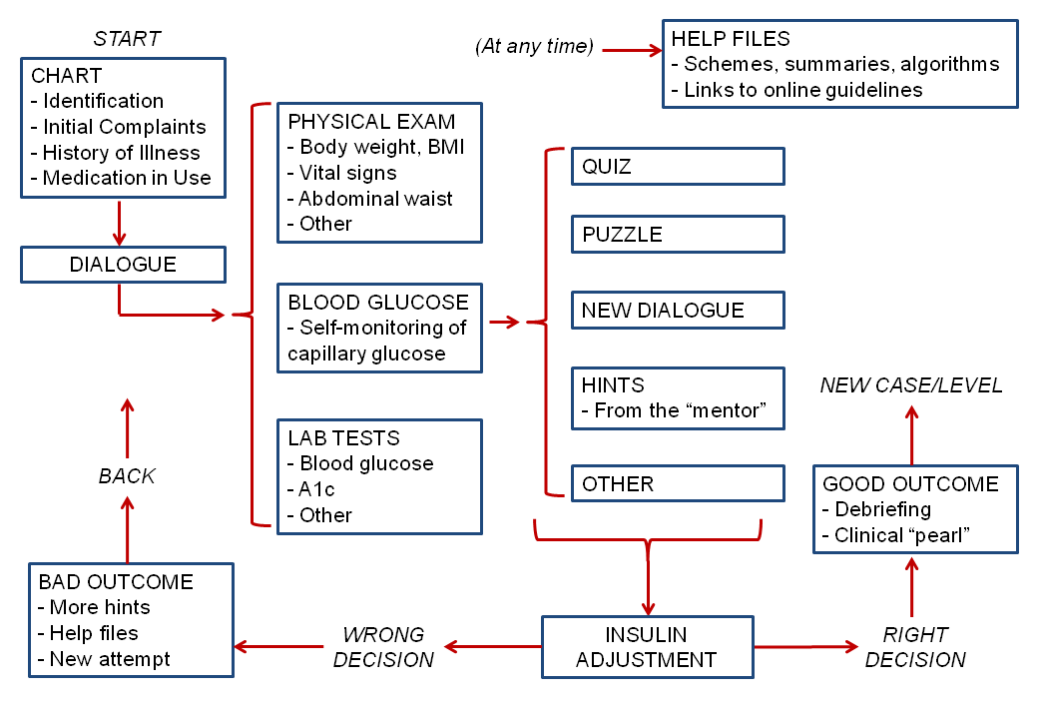
Basic structure of an InsuOnline level.

We created 16 diabetic patients, reflecting common clinical scenarios in actual primary health care clinical practice, each one corresponding to at least one of the topics of game´s minimum curriculum. Each game level has a new patient presented to the player. The patients/levels are disposed in order of increasing complexity. The player´s avatar must make decisions on the best therapeutic option to improve each patient´s glycemic control at every level. Right decisions lead to progression to the next level, and incorrect choices lead to a new attempt at the same level. The basic structure of each level is shown in Figure 1.

### Interactive Components and Game Elements

Several ways of interaction and game elements were included to create a pleasant experience and to maximize players´ intrinsic motivation. The player can read patients´ clinical charts, dialogue with them (be choosing among a few options of "talks"), see their lab tests and fingertip glucose readings, review their physical exam, answer quiz challenges, solve puzzles, get tips from the mentor and a nurse, and, prescribe insulin. Sound track, score, visual and sound effects, patients' mood (the patient gets angry if the player makes too many errors), and between-levels animation scenes (machinimas) were also included, to improve the gaming experience and players´ motivation.

### Pedagogical Elements

Several pedagogical elements were included in the game, aiming for the best educational effects. These were based on the principles of adult learning and problem-based learning, including motivation, goal-orientation, relevancy-orientation, self-pacing, timely and appropriate feedback, reinforcement of learning, informal environment, contextualization, and practical (ie, hands-on) approach with active participation of the learner [27,28].

The game gives immediate feedback (presented by the mentor character), comparing player’s decisions with recommendations from clinical guidelines [1,29-31]. Correct decisions lead to gaining points and progression in the game. At every step, the game offers additional learning resources: help files (eg, algorithms, summaries, and clinical pearls), orientation from the mentor, and links to bibliographic references.

### Usability and Playability Evaluation

The next step will be the usability and playability assessments. For this, we will enroll 4 physicians who work in Londrina, Brazil (2 female and 2 male; 2 with gaming/computer previous experience and 2 without experience) and 4 undergraduate medical students from Universidade Estadual de Londrina (UEL) (2 female and 2 male, with and without gaming experience), who will play the game on a desktop computer, in a controlled environment, each participant alone in a single session. Usability data will be assessed using Web-based System Usability Scale (SUS), as previously described by Brooke [32], and playability will be assessed by Web-based Heuristic Evaluation for Playability, as described by Desurvire et al [33] The actions of the players will be recorded by the software for further analysis by the researchers (LAD, RMS). Further adjustments will be made in the game, according to responses obtained in this phase of the study.

### Educational Efficacy Assessment

After final adjustments in the game, guided by usability and playability assessments, the efficacy of InsuOnline as an educational tool will be assessed in an unblinded randomized controlled trial [34]. We will send a letter of invitation to all primary care physicians (PCPs) who work in Londrina to participate in the study. If those PCPs do not fulfill our sample size, we will also invite PCPs from other cities in the state of Paraná (Brazil), such as Maringá, Curitiba, or São José dos Pinhais. The PCPs who are willing to participate in the study will be included and randomized at study entry, using a random number generator, to 1 of 2 groups. We will exclude clinical endocrinologists or diabetologists.

Physicians enrolled in the Group A will be exposed to InsuOnline. They will be asked to play the game until its end, on the Web (with an individual login) in their own time and rhythm. Physicians enrolled in the Group B will undertake a traditional instructional session, during one afternoon, composed of a short lecture and a group discussion of clinical cases, which will be identical to the ones included in InsuOnline. This traditional instructional session will focus on the same teaching topics of the game, and it will be coordinated by a clinical endocrinologist not linked to the research team in order to avoid potential biases. This endocrinologist will be trained by the researcher endocrinologists, and he will use didactic material prepared by our team.

We will evaluate subjects´ knowledge on insulin therapy using a Web-based questionnaire, containing 10-20 multiple choice questions. The questions will be clinical vignettes of diabetic patients who require initiation and/or adjustment of insulin. For each question, the participant should choose the best option for achieving a better glycemic control, according to current guidelines [1,29-31. Questions regarding insulin therapy will be selected from the American Diabetes Association Self-Assessment Program, Module 2 (Pharmacological Treatment of Hyperglycemia) [35], translated to Portuguese, and adapted to be compatible with the game´s clinical scenarios and learning objectives. Participants´ attitudes regarding diabetes will also be assessed, by the application of Web-based Diabetes Attitudes Scale, version 3 (DAS-3) [36]. The questionnaire and the DAS-3 will be answered by the participants at 3 time points: before the intervention (pre-test), immediately after the intervention (after-test), and 6 months after the intervention (late-test). The average number of right answers will be presented and compared in the different time points within each group (intragroup) using analysis of variance (ANOVA), and between the 2 groups at each time point (intergroup) using Student´s *t* test. A bicaudal significance level of 0.05 will be adopted. Data analysis will be done by intention-to-treat, with last observation carried forward, which means that all randomized participants´ data will be included in the final analysis.

The participants will be asked a few Likert-scale and free text questions at the end of each intervention to assess their general impressions about the intervention (mostly to assess if it was pleasant or enjoyable). Software-recorded data on player´s usage of the game will be analyzed to assess the number of logins, time spent on the game, etc. For the participants in Group A that do not finish the game, the reasons for early withdrawal will be collected by an online questionnaire. We will contact (by phone or email) the participants who do not eventually fulfill the questionnaires at least twice.

In order to detect a minimum standard deviation of 0.5 on the average of right answers with 80% of statistical power at 5% of significance level, we will need to include 128 subjects in our study (64 in each group). We will enroll 160 subjects (80 in each group), in order to compensate for an expected 25% dropout rate.

### Ethical Considerations

Participation will be anonymous and voluntary, and all subjects will be asked to provide written informed consent, according to Brazilian Health Ministry´s regulations (Multimedia Appendix 1). The study protocol was approved by our local Research Ethics Committee (UEL, #051/2011 and #051/2012), and registered by UEL research board (Research Project #07471).

## Results

InsuOnline is currently at the final stages of development and programming of its alpha version. The research team is currently playing and testing this version of the software, in order to detect and correct occasional bugs or problems.

**Figure 2:**
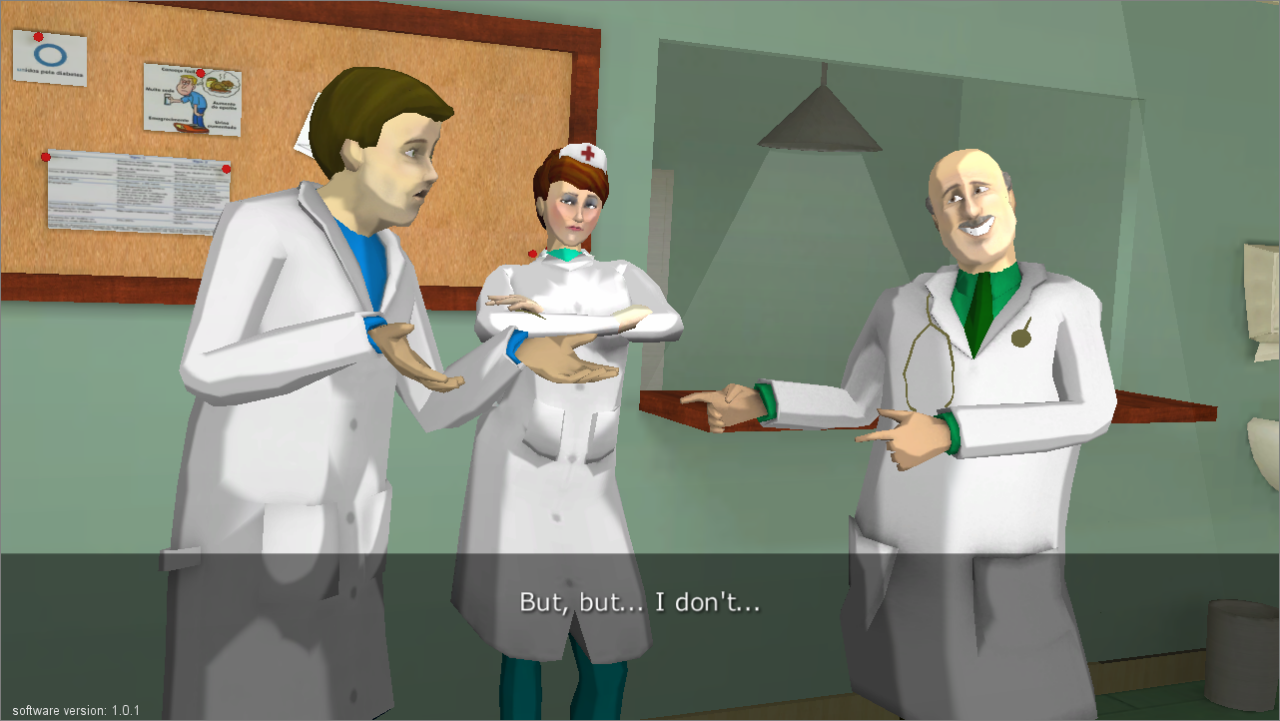
The main characters: the young doctor (player´s avatar), the nurse, and the older doctor (mentor).

**Figure 3:**
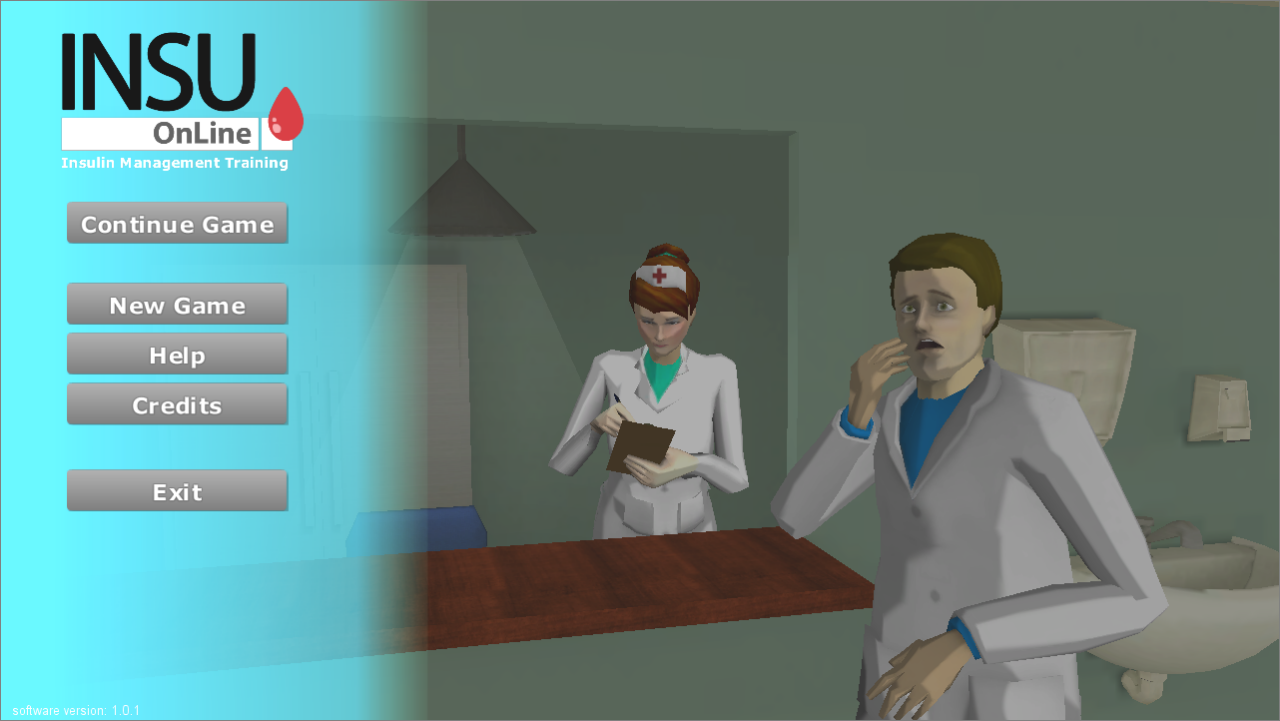
InsuOnline main menu.

**Figure 4:**
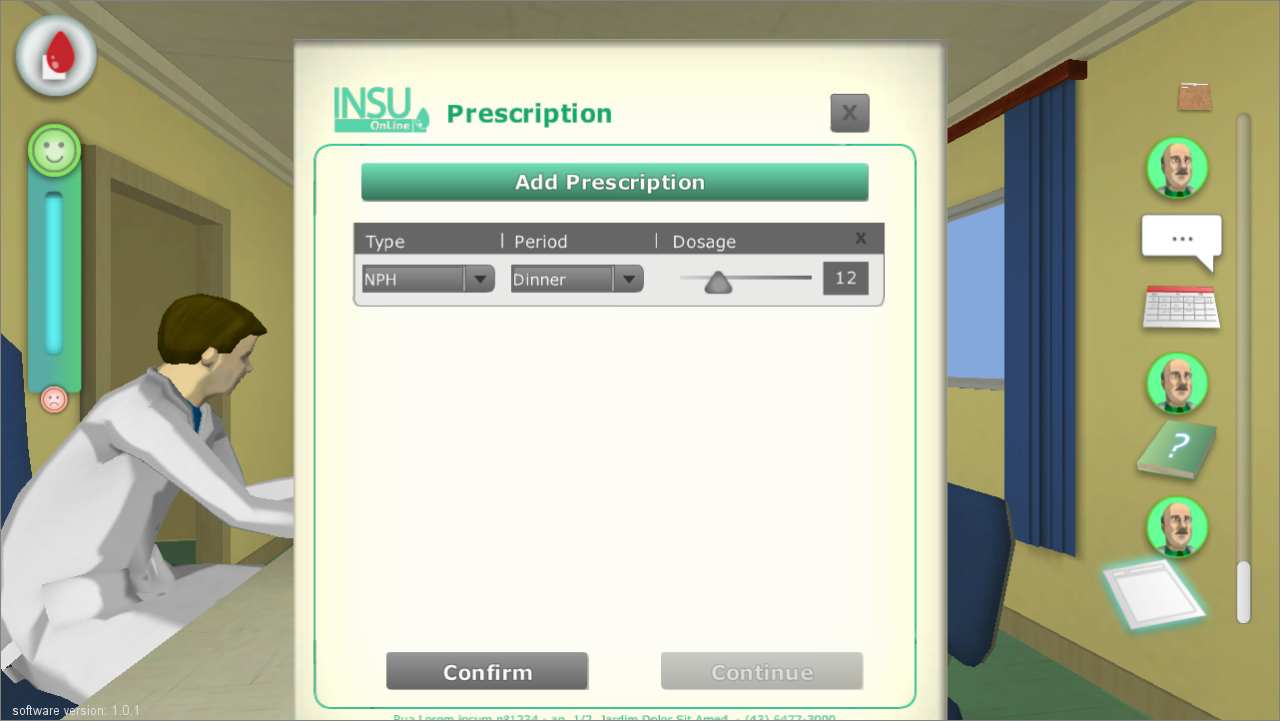
Panel for insulin prescription.

**Figure 5:**
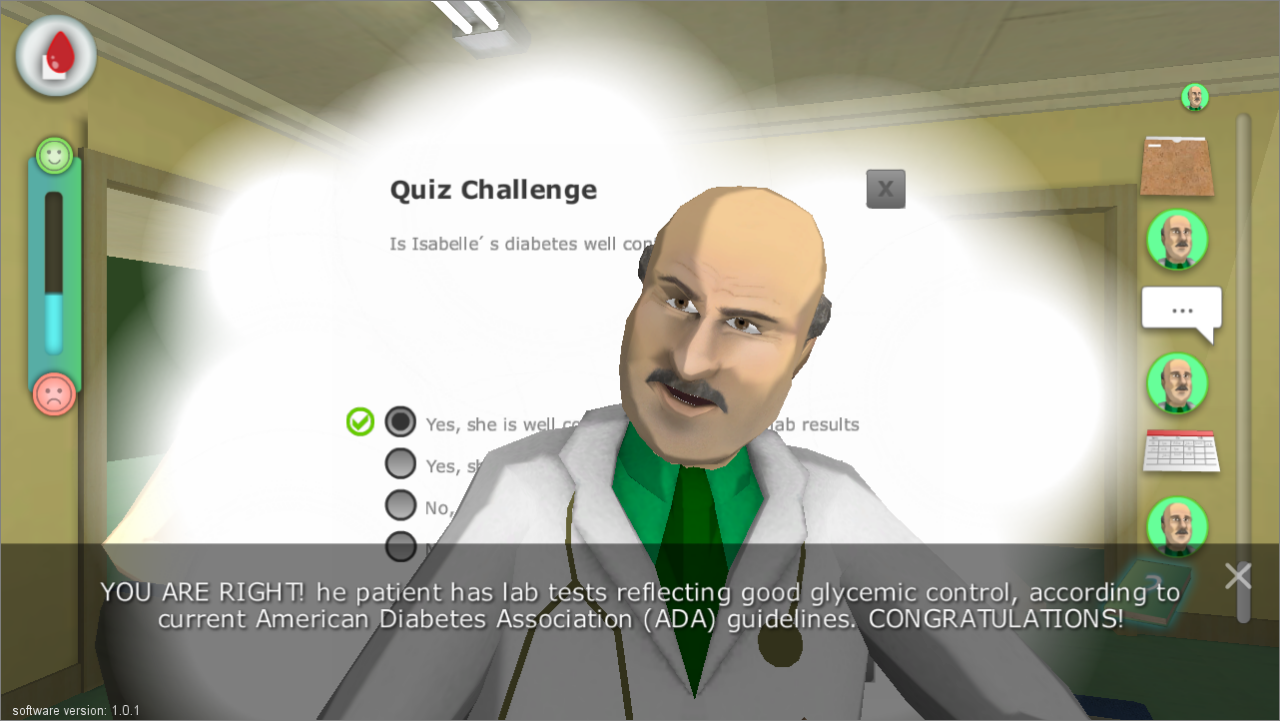
Feedback from the mentor after a player´s action.

The adventure genre was chosen, since it allows the learner to explore and try to solve consecutive problems, a design most compatible with adult education principles [11]. The game is represented in 3D, with third-person vision, representing the behavior of the character at scene. The characters were designed to present a visual appearance that slightly resembles those in the classic game "The Sims" [37], in that they were not too serious/realistic or cartoon-like. A few screenshots of the game can be seen in the Figures 2 to 5.

The programming was done using the game engine Unity, and the 3D-Suite Blender was used for the character modeling and animation. Players´ interaction with the game is made via mouse.

## Discussion

Games and simulators are useful for health care professionals´ education on various fields [38]. Specifically regarding diabetes education, a few simulators are available for clinicians to learn and practice insulin prescribing. These have been well accepted by the target audience, but lack an objective measurement of their educational efficacy [22-25]. However in our opinion, these tools have few ways of interaction and are excessively text-based. As a result, the existing simulators are not very attractive and probably unsuccessful for arousing the intrinsic motivation of their audience, who will often need some external stimuli in order to play them.

In order to fulfill learning purposes, any intervention must incorporate the main principles of adult education**:** individualized, self-pacing, contextualized learning, with active experimentation and appreciation of previous knowledge [12,27,39]. The inclusion of game elements adds entertainment to the learning experience, increasing the intrinsical motivation of the learner to practice and to learn, making the learning experience more enjoyable and potentially more effective [39]. For these reasons, games can be a more interesting and successful approach to CME than simulators and other forms of distance education, such as virtual world interventions [40] or telemedicine consultations [41].

Many games have been developed for education of diabetic patients showing good results, including reducing the rates of urgent hospitalization of diabetic children, for example [42]. However to our knowledge, there were no reported games for education of medical doctors on insulin or DM, and this is the first report of a game oriented to education of primary care physicians on insulin prescription (initiation, adjustment, and problem solving). The need of a new approach for education of health professionals regarding insulin therapy is justified by their difficulties in this field. In Londrina, 87% of PCPs directly involved in the treatment of diabetic patients reported at least one difficulty or insecurity with insulin use, and 38% admitted that they would not initiate insulin for a hypothetical patient with type 2 diabetes who was clearly in need of insulin initiation [43].

Also, this is the first known research trial studying the educational efficacy of a computer game in teaching PCPs about insulin therapy for DM. This is important, as most games for health care have not been validated as tools for education or health outcomes improvement, and there is a great need of good quality studies which can provide good scientific evidences to support the fast-growing field of games for health. Therefore, our study was designed to follow the rigorous guidelines for research on games´ effectiveness proposed by Kato [44].

We hope to demonstrate that a game can be an attractive and effective option for CME on insulin and diabetes. If we can do that, it would give more support to the idea that games can be very good options for large-scale CME, since they can be published on the Web to reach more health professionals compared to traditional learning activities, such as conferences or symposia, which cost substantial amount of time and money. A well-designed Web-based learning game can be used by the learners in their own time and rhythm, at possibly smaller costs, and have a number of significant potential advantages.

In conclusion, we demonstrated that the design and development of a game for education of PCPs on insulin management is possible with the collaboration of a multidisciplinary team. Although its efficacy still needs further evaluation, we think that InsuOnline can be a valuable tool for large-scale CME on DM, in view of its easy dissemination on the Web, customizable content, and accordance with adult learning principles. We hope it can contribute to improving PCPs´ knowledge and optimize DM control in primary care.

## Multimedia Appendix 1

Informed Consent Form [in Portuguese].

## Multimedia Appendix 2

CONSORT EHEALTH Checklist V1.6.2 [34].

## Abbreviations

A1c: glycated hemoglobin A1c
BMI: body mass index
CME: continuing medical education
DM: diabetes mellitus
PCP: primary care physicians
UEL: Universidade Estadual de Londrina
