# Supplementary material for: InsuOnline, a Serious Game to Teach Insulin Therapy to Primary Care Physicians: Design of the Game and a Randomized Controlled Trial for Educational Validation
Source: JMIR Res Protoc. 2013 Jan 21;2(1):e5. doi: 10.2196/resprot.2431 (PMC3628160; doi:10.2196/resprot.2431)
Supplement: Supplementary file 1 [file resprot_v2i1e5_app1.pdf]

Universidade Estadual de Londrina / Centro de Ciências da Saúde

Termo de Consentimento Livre e Esclarecido

Título da pesquisa:
"Desenvolvimento e Avaliação de um *Serious Game* Baseado na *Web* para Treinamento de Profissionais de Saúde no Manejo da Insulina no Tratamento do Diabetes Mellitus**"**

Prezado(a) Senhor(a):

Gostaríamos de convidá-lo a participar da pesquisa **"**Desenvolvimento e Avaliação de um *Serious Game* Baseado na *Web* para Treinamento de Profissionais de Saúde no Manejo da Insulina no Tratamento do Diabetes Mellitus**"**, realizada no Centro de Ciências da Saúde da Universidade Estadual de Londrina**.**

O objetivo da pesquisa é avaliar se o uso de um jogo de computador pode ser tão útil quanto o uso de aulas convencionais, para o treinamento de médicos e estudantes de Medicina no uso de insulina para o tratamento de pacientes com diabetes mellitus**.**

A sua participação é muito importante, e ela se daria da seguinte forma: você seria sorteado para assistir à aula OU participar do jogo de computador. Antes e depois da sua participação em uma dessas atividades, você seria submetido a um teste para avaliar o seu nível de conhecimento e suas atitudes sobre o uso de insulina no tratamento do diabetes. Um novo teste também seria agendado para 6 meses depois da sua participação em uma dessas atividades, para avaliar se você ainda guarda os mesmos conhecimentos que adquiriu logo após a aula ou o uso do jogo de computador sobre o assunto.

Gostaríamos de esclarecer que sua participação é totalmente voluntária, podendo você recusar-se a participar, ou mesmo desistir a qualquer momento, sem que isto acarrete qualquer ônus ou prejuízo à sua pessoa. Informamos ainda que as informações serão utilizadas somente para os fins desta pesquisa e serão tratadas com o mais absoluto sigilo e confidencialidade, de modo a preservar a sua identidade.

Após o final da análise dos dados, todas as provas que você fez serão destruídas, assim como os registros do jogo de computador que mostram a sua participação serão apagados.

Os benefícios esperados são: provar que o uso de um jogo de computador pode ser uma estratégia educacional tão boa quanto aulas convencionais, para ensinar aspectos práticos do uso de insulina no tratamento do diabetes. Com isso, esperamos contribuir para otimizar o treinamento de médicos e estudantes de Medicina no tratamento do diabetes e, assim, melhorar a qualidade do atendimento prestado à população diabética.

Não há riscos associados à sua participação nesta pesquisa, além da necessidade de dedicar algum tempo às aulas ou ao jogo, e também às provas de avaliação antes e após essas atividades.

Informamos que o senhor não pagará nem será remunerado por sua participação. Garantimos, no entanto, que todas as despesas decorrentes da pesquisa serão ressarcidas, quando devidas e decorrentes especificamente de sua participação na pesquisa.

Caso você tenha dúvidas ou necessite de maiores esclarecimentos pode contatar o pesquisador responsável: Dr. Leandro Arthur Diehl, fone (43) 3371-2234 ou (43) 3371-2218, email: drgaucho@yahoo.com, ou procurar o Comitê de Ética em Pesquisa Envolvendo Seres Humanos da Universidade Estadual de Londrina, na Avenida Robert Kock, nº 60, ou no telefone 3371-2490.

Este termo deverá ser preenchido em duas vias de igual teor, sendo uma delas, devidamente preenchida e assinada, entregue a você.

Londrina, ___ de _____________de 2013.

Dr. Leandro A. Diehl - Pesquisador Responsável

Eu, ____ ,
tendo sido devidamente esclarecido sobre os procedimentos da pesquisa, concordo em participar **voluntariamente** da pesquisa descrita acima.

Assinatura:_____________________________

Data:___________________
