# Supplementary material for: InsuOnline, a Serious Game to Teach Insulin Therapy to Primary Care Physicians: Design of the Game and a Randomized Controlled Trial for Educational Validation
Source: JMIR Res Protoc. 2013 Jan 21;2(1):e5. doi: 10.2196/resprot.2431 (PMC3628160; doi:10.2196/resprot.2431)
Supplement: Supplementary file 2 [file resprot_v2i1e5_app2.pdf]

CONSORT-EHEALTH Checklist V1.6.2 Report

(based on CONSORT-EHEALTH V1.6), available at [http://tinyurl.com/consort-ehealth-v1-6].

2431

Date completed

12/21/2012 14:54:03

by

Leandro Diehl

InsuOnline©: a serious game to teach insulin therapy to primary care physicians: design of a randomized controlled trial for educational validation

TITLE

1a-i) Identify the mode of delivery in the title

The game addressed in this study will be delivered to the participants via web for the randomized trial on educational efficacy, but it will be installed on a desktop for prior tests of usability and playability. The title is already too long and we think that the mode of delivery is not so relevant, in this case, to justify its addition to the title. However, this information is presented in the abstract.

1a-ii) Non-web-based components or important co-interventions in title

The game (study) group will not be exposed to any non-web-based componentes, during the main phase of the randomized controlled trial.

1a-iii) Primary condition or target group in the title

"Primary care physicians"

ABSTRACT

1b-i) Key features/functionalities/components of the intervention and comparator in the METHODS section of the ABSTRACT

"InsuOnline© is a serious game which includes game elements and a well-defined, evidence-based curriculum of topics on insulin therapy. InsuOnline© is an Adventure game, with clinical cases (levels) disposed in increasing complexity. The player needs to assess each case and make decisions on insulin initiation or adjustment. In every step, the player receives immediate feedback, based on main guidelines on the subject, and has access to help files.."

1b-ii) Level of human involvement in the METHODS section of the ABSTRACT

There is human involvement only in the control (comparator) group, exposed to traditional educational activities: "traditional teaching activities (lecture and group discussion)".

1b-iii) Open vs. closed, web-based (self-assessment) vs. face-to-face assessments in the METHODS section of the ABSTRACT

"Knowledge on how to initiate and adjust insulin will be assessed by a web-based multiple-choice questionnaire, and attitudes regarding diabetes/insulin will be assessed by Diabetes Attitude Scale 3, at 3 time points: before, immediately after, and 6 months after the intervention."

1b-iv) RESULTS section in abstract must contain use data

The manuscript is a description of a research protocol, and does not have any results of the study yet. So, the Results section only includes the description of the game.

1b-v) CONCLUSIONS/DISCUSSION in abstract for negative trials

There are not any results yet, since the manuscript describes a research protocol.

INTRODUCTION

2a-i) Problem and the type of system/solution

"Continuing medical education (CME) on DM and insulin is often advocated as a solution to optimize the knowledge and the practice of PCPs [9]; however, traditional CME activities (such as lectures and group discussions) have small and short-lasting efficacy [10]. Thus, new educational methods are urgently required."

2a-ii) Scientific background, rationale: What is known about the (type of) system

"Digital games are currently one of the six greatest trends in higher education [11], since they are able to "create a tight marriage among content, game play, and valued ways of thinking and acting" [12]. One of the reasons for the increasing interest in games for higher and professional education is the huge familiarity of most today´s college students (the "digital natives") with the medium [13]. Most medical students, for instance, even those who do not play video games, have highly favorable views about the use of video games and new technologies in medical education [14]. However, the most compelling reason for adopting learning games, probably, is their pedagogical adequacy, since good learning games are usually built by the same rules who guide the design of effective learning activities: stimulus to players´ intrinsic motivation, practice and repetition, effective feedback, arousal of positive feelings, intensity of the experience, and learner choice/involvement [15]. In the medical area, particularly, the use of games and simulators for learning clinical skills has the additional advantage of increasing the safety of real patients [16]."

METHODS

3a) CONSORT: Description of trial design (such as parallel, factorial) including allocation ratio

"We report the design and development of a PC serious game for teaching PCPs about initiation and adjustment of insulin in the treatment of DM, and describe the design of a randomized controlled trial to assess if the game can be educationally effective."

3b) CONSORT: Important changes to methods after trial commencement (such as eligibility criteria), with reasons

The trial has not started yet, so no changes in the methods were made until this point.

3b-i) Bug fixes, Downtimes, Content Changes

"After final adjustments in the game, guided by usability and playability assessments,..."

4a) CONSORT: Eligibility criteria for participants

"We will invite all primary care physicians (PCPs) who work in the city of Londrina to participate in the study; if those PCPs do not fulfill our sample size, we will also invite PCPs from other cities of the state of Paraná (Brazil), such as Maringá, Curitiba, or São José dos Pinhais. The PCPs which are willing to participate in the study will be included and randomized at study entry, to one of 2 groups. We will exclude clinical endocrinologists or diabetologists.
"

4a-i) Computer / Internet literacy

This issue was addressed for the usability/playability assessments, but for the next phase (educational efficacy assessment), we will include all PCPs from the city of Londrina who accept the invitation. This sample will probably include people with different degrees of computer literacy, and it will probably be representative of the population of Brazilian PCPs.

4a-ii) Open vs. closed, web-based vs. face-to-face assessments:

Recruitment: "We will send a letter of invitation to all primary care physicians (PCPs) who work in the city of Londrina to participate in the study..."
Intervention: "Physicians enrolled in the Group A will be exposed to InsuOnline©. They will be asked to play the game until its end, on the web or installed on their desktops, in their own time and rhythm. Physicians enrolled in the Group B will undertake a presential learning session, during one afternoon, composed by a short lecture and a group discussion of clinical cases identical to the ones included in InsuOnline©."
Assessment: "We will evaluate subjects´ knowledge on insulin therapy using a web-based questionnaire, containing 10 to 20 multiple-choice questions. The questions will be clinical vignettes of diabetic patients who require initiation and/or adjustment of insulin; for each, the participant should choose the best option for achieving a better glycemic control, according to current guidelines. Questions regarding insulin therapy will be choosen from the American Diabetes Association Self-Assessment Program, Module 2 (Pharmacological Treatment of Hyperglycemia), translated to Portuguese, and adapted to be compatible with the game´s clinical scenarios and learning objectives. Participants´ attitudes regarding diabetes will also be assessed, by the application of web-based Diabetes Attitudes Scale, version 3 (DAS-3)."

4a-iii) Information giving during recruitment

Informed consent form was attached as an Appendix.
"Participation will be anonymous and voluntary, and all subjects will previously provide written informed consent, according to Brazilian Health Ministry´s regulations (see the informed consent form in the Appendix). "

4b) CONSORT: Settings and locations where the data were collected

"Usability data will be assessed using web-based System Usability Scale (SUS), as previously described by Brooke, and playability will be assessed by web-based Heuristic Evaluation for Playability (HEP), as described by Desurvire et al."

4b-i) Report if outcomes were (self-)assessed through online questionnaires

"Usability data will be assessed using web-based System Usability Scale (SUS), as previously described by Brooke, and playability will be assessed by web-based Heuristic Evaluation for Playability (HEP), as described by Desurvire et al. The actions of the players will be recorded by the software for further analysis by the researchers (LAD, RMS). "

4b-ii) Report how institutional affiliations are displayed

Not relevant, since most PCPs already know the main researcher (LAD).

5) CONSORT: Describe the interventions for each group with sufficient details to allow replication, including how and when they were actually administered

5-i) Mention names, credential, affiliations of the developers, sponsors, and owners

"InsuOnline© design and development was entirely founded by personal resources from the authors LAD, RMS, JBA, PAG, who are copyright holders. All authors contribute to design and evaluation of the game."

5-ii) Describe the history/development process

"InsuOnline© is currently at the final stages of development and programming of its alpha version. The research team is playing and testing this version of the software, in order to detect and correct occasional bugs or problems."

"Usability and Playability Evaluation
The next step will be usability and playability assessment. For this, we will enroll 4 physicians who work in the city of Londrina (2 female and 2 male; 2 with gaming/computer previous experience and 2 without experience) and 4 undergraduate medical students from UEL (2 female and 2 male, with and without gaming experience), whom will play the game on a desktop, in a controlled environment (lab for usability tests), each participant alone in a single session. Usability data will be assessed using web-based System Usability Scale (SUS), as previously described by Brooke [BROOKE 1986], and playability will be assessed by web-based Heuristic Evaluation for Playability (HEP), as described by Desurvire et al.[13] The actions of the players will be recorded by the software for further analysis by the researchers (LAD, RMS). Further adjustments will be made in the game, according to responses obtained in this phase of the study."

5-iii) Revisions and updating

The trial has not started yet, so no important changes were made to the original software (in final phase of development) until this point.

5-iv) Quality assurance methods

5-v) Ensure replicability by publishing the source code, and/or providing screenshots/screen-capture video, and/or providing flowcharts of the algorithms used

"A few screenshots of the game can be seen in the Figures 3 to 6. "

5-vi) Digital preservation

"A few screenshots of the game can be seen in the Figures 3 to 6. "

5-vii) Access

"Physicians enrolled in the Group A will be exposed to InsuOnline©. They will be asked to play the game until its end, on the web (with an individual login), in their own time and rhythm. "

5-viii) Mode of delivery, features/functionalities/components of the intervention and comparator, and the theoretical framework

"Level Design
Sixteen diabetic patients were created, reflecting common clinical scenarios in actual primary health care clinical practice, each one corresponding to at least one of the topics of game´s minimum curriculum. Each patient is a game level. The patients/levels are disposed in an increasing complexity order. The player´s avatar must take decisions on which is the best therapeutic option to improve each patient´s glycemic control. Right decisions lead to progression to the next level. The basic structure of each case (level) is exposed in Figure 1.

Interaction Ways and Game Elements
Players´ interaction with the game is made via mouse. Several ways of interaction and game elements were included along the game, to make it a pleasant experience and to maximize players´ intrinsic motivation. The player can read patients´s clinical charts, dialogue with them (choosing among a few options of "talks"), see their lab tests or fingertip glucose readings, review their physical exam, answer to "quiz challenges", solve puzzles, get "tips" from the mentor and from a nurse, and, finally, prescribe insulin. Soundtrack, score, visual and sound effects, patients' "mood" (the patient gets angry if the player makes too many errors), and between-levels animation scenes (machinimas) were also included, to improve gaming experience and players´ motivation.

Pedagogical Elements
Several pedagogical elements were included in the game, aiming for the best educational effects, based on principles of Adult Learning and Problem-Based Learning: motivation, goal-orientation, relevancy-orientation, self-pacing, timely and appropriate feedback, reinforcement of learning, informal environment, contextualisation, and practical ("hands-on") approach with active participation of the learner [27-28].
The game gives immediate feedback (given by the character of the "mentor"), comparing player's decisions with recommendations from clinical guidelines [1,29-31]. Correct decisions lead to gaining points and progression in the game. At every step, the game offers additional learning resources: PDF-format help files (algorithms, summaries, clinical pearls), orientation from a "mentor" character, and links to bibliographic references. If the player fails to make the right choices, he can try the same level again.
"

5-ix) Describe use parameters
"Physicians enrolled in the Group A will be exposed to InsuOnline©. They will be asked to play the game until its end, on the web (with an individual login), in their own time and rhythm. "
5-x) Clarify the level of human involvement
Human involvement will be present only in the control (comparator) group.
5-xi) Report any prompts/reminders used
The game will be relatively short, so we think prompts or reminders will not be necessary.
5-xii) Describe any co-interventions (incl. training/support)
No co-interventions will be made in the game group.
Traditional activities of continued medical education will be undertaken by the control group.
6a) CONSORT: Completely defined pre-specified primary and secondary outcome measures, including how and when they were assessed
"We will evaluate subjects´ knowledge on insulin therapy using a web-based questionnaire, containing 10 to 20 multiple-choice questions. The questions will be clinical vignettes of diabetic patients who require initiation and/or adjustment of insulin; for each, the participant should choose the best option for achieving a better glycemic control, according to current guidelines. Questions regarding insulin therapy will be choosen from the American Diabetes Association Self-Assessment Program, Module 2 (Pharmacological Treatment of Hyperglycemia) [35], translated to Portuguese, and adapted to be compatible with the game´s clinical scenarios and learning objectives. Participants´ attitudes regarding diabetes will also be assessed, by the application of web-based Diabetes Attitudes Scale, version 3 (DAS-3). [36] A few free-text questions will also be asked to the participants at the end of each intervention, in order to assess their general impressions about the intervention (mostly to assess if it was pleasant or enjoyable)."
6a-i) Online questionnaires: describe if they were validated for online use and apply CHERRIES items to describe how the questionnaires were designed/deployed
The questionnaires used in this study were adequately validated by previous research, except for the free-text questions on general impressions about the intervention.
6a-ii) Describe whether and how “use” (including intensity of use/dosage) was defined/measured/monitored
"Software-recorded data on player´s usage of the game will be analyzed to assess: number of logins, time spent on the game, etc."
6a-iii) Describe whether, how, and when qualitative feedback from participants was obtained
"Software-recorded data on player´s usage of the game will be analyzed to assess: number of logins, time spent on the game, etc."
6b) CONSORT: Any changes to trial outcomes after the trial commenced, with reasons
The trial has not started yet, so no changes in the methods were made until this point.
7a) CONSORT: How sample size was determined
7a-i) Describe whether and how expected attrition was taken into account when calculating the sample size
"In order to detect a minimum difference of 0.5 standard deviation on the average of right answers, with 80% of statistical power, at 5% of significance level, we will need to include 128 subjects in our study (64 in each group). "
7b) CONSORT: When applicable, explanation of any interim analyses and stopping guidelines
No interim analyses will be undertaken.
8a) CONSORT: Method used to generate the random allocation sequence
"The PCPs which are willing to participate in the study will be included and randomized at study entry, using a random number generator, to one of 2 groups. "
8b) CONSORT: Type of randomisation; details of any restriction (such as blocking and block size)
Simple randomization.
9) CONSORT: Mechanism used to implement the random allocation sequence (such as sequentially numbered containers), describing any steps taken to conceal the sequence until interventions were assigned
"The PCPs which are willing to participate in the study will be included and randomized at study entry, using a random number generator, to one of 2 groups. "
10) CONSORT: Who generated the random allocation sequence, who enrolled participants, and who assigned participants to interventions
"The PCPs which are willing to participate in the study will be included and randomized at study entry, using a random number generator, to one of 2 groups. "
11a) CONSORT: Blinding - If done, who was blinded after assignment to interventions (for example, participants, care providers, those assessing outcomes) and how
11a-i) Specify who was blinded, and who wasn’t
"After final adjustments in the game, guided by usability and playability assessments, the efficacy of InsuOnline© as an educational tool will be assessed in an unblinded randomized controlled trial (Figure 2)"
11a-ii) Discuss e.g., whether participants knew which intervention was the “intervention of interest” and which one was the “comparator”

11b) CONSORT: If relevant, description of the similarity of interventions
"Physicians enrolled in the Group A will be exposed to InsuOnline©. They will be asked to play the game until its end, on the web (with an individual login), in their own time and rhythm. Physicians enrolled in the Group B will undertake a presential learning session, during one afternoon, composed by a short lecture and a group discussion of clinical cases identical to the ones included in InsuOnline©. This presential session will focus on the same teaching topics of the game, and it will be coordinated by a clinical endocrinologist not vinculated to the research team, in order to avoid potential biases. This endocrinologist will be previously trained by the researcher endocrinologists, and he will use didactic material prepared by our team. "
12a) CONSORT: Statistical methods used to compare groups for primary and secondary outcomes
"The questionnaire and the DAS-3 will be answered by the participants in 3 time points: before the intervention (pre-test), immediately after the intervention (after-test), and 6 months after the intervention (late-test). The average of right answers will be presented and compared: a) in the different time points, within each group (intragroup), using analysis of variance (ANOVA); and b) between the two groups, at each time point (intergroup), using Student´s t test. A bicaudal significance level of 0,05 will be adopted. "
12a-i) Imputation techniques to deal with attrition / missing values
"Data analysis will be done by intention-to-treat. "
12b) CONSORT: Methods for additional analyses, such as subgroup analyses and adjusted analyses
No subgroup analyses will be made.

RESULTS

13a) CONSORT: For each group, the numbers of participants who were randomly assigned, received intended treatment, and were analysed for the primary outcome
We do not have these results yet.
13b) CONSORT: For each group, losses and exclusions after randomisation, together with reasons
We do not have these results yet.

13b-i) Attrition diagram
We do not have these results yet.
14a) CONSORT: Dates defining the periods of recruitment and follow-up
It will be done probably in the first semester of 2013, but we still depend on the end of the development and review of the software.
14a-i) Indicate if critical “secular events” fell into the study period
Not relevant in this case.
14b) CONSORT: Why the trial ended or was stopped (early)
We do not have any results yet.
15) CONSORT: A table showing baseline demographic and clinical characteristics for each group
We do not have these results yet, but it will be presented.
15-i) Report demographics associated with digital divide issues
We do not have these results yet, but it will be presented.
16a) CONSORT: For each group, number of participants (denominator) included in each analysis and whether the analysis was by original assigned groups
16-i) Report multiple “denominators” and provide definitions
We do not have these results yet, but it will be presented.
16-ii) Primary analysis should be intent-to-treat
"Data analysis will be done by intention-to-treat. "
17a) CONSORT: For each primary and secondary outcome, results for each group, and the estimated effect size and its precision (such as 95% confidence interval)
"In order to detect a minimum difference of 0.5 standard deviation on the average of right answers, with 80% of statistical power, at 5% of significance level, we will need to include 128 subjects in our study (64 in each group). "
We do not have any results so far.
17a-i) Presentation of process outcomes such as metrics of use and intensity of use
We do not have any results yet.
17b) CONSORT: For binary outcomes, presentation of both absolute and relative effect sizes is recommended
"The average of right answers will be presented and compared: a) in the different time points, within each group (intragroup), using analysis of variance (ANOVA); and b) between the two groups, at each time point (intergroup), using Student´s t test. "
We do not have any results yet.
18) CONSORT: Results of any other analyses performed, including subgroup analyses and adjusted analyses, distinguishing pre-specified from exploratory
No subgroup analyses will be made.
18-i) Subgroup analysis of comparing only users
No subgroup analyses will be made.
19) CONSORT: All important harms or unintended effects in each group
"A few free-text questions will also be asked to the participants at the end of each intervention, in order to assess their general impressions about the intervention (mostly to assess if it was pleasant or enjoyable). "
No significant risks will be taken by the participants.
19-i) Include privacy breaches, technical problems
We do not have these results yet.
19-ii) Include qualitative feedback from participants or observations from staff/researchers
"A few free-text questions will also be asked to the participants at the end of each intervention, in order to assess their general impressions about the intervention (mostly to assess if it was pleasant or enjoyable). "

DISCUSSION

20) CONSORT: Trial limitations, addressing sources of potential bias, imprecision, multiplicity of analyses
20-i) Typical limitations in ehealth trials
It will be discussed, but we do not have any results yet.
21) CONSORT: Generalisability (external validity, applicability) of the trial findings
21-i) Generalizability to other populations
It will be discussed, but we do not have any results yet.
21-ii) Discuss if there were elements in the RCT that would be different in a routine application setting
It will be discussed, but we do not have any results yet.
22) CONSORT: Interpretation consistent with results, balancing benefits and harms, and considering other relevant evidence
22-i) Restate study questions and summarize the answers suggested by the data, starting with primary outcomes and process outcomes (use)
We do not have any results yet.
22-ii) Highlight unanswered new questions, suggest future research
It will be discussed, but we do not have any results yet.

Other information

23) CONSORT: Registration number and name of trial registry
Clinicaltrials.gov: NCT01759953. Universidade Estadual de Londrina, Research
24) CONSORT: Where the full trial protocol can be accessed, if available
https://www.sistemasweb.uel.br/system/prj/pes/pdf/pes_pesquisa_07471.pdf
25) CONSORT: Sources of funding and other support (such as supply of drugs), role of funders
"InsuOnline© design and development was entirely founded by personal resources from the authors LAD, RMS, JBA, PAG, who are copyright holders. All authors contribute to design and evaluation of the game."
X26-i) Comment on ethics committee approval
"Participation will be anonymous and voluntary, and all subjects will previously provide written informed consent, according to Brazilian Health Ministry´s regulations (see the informed consent form in the Appendix). The study protocol was approved by our local Research Ethics Committee (UEL, #051/2011 and #051/2012), and registered by UEL research board (Research Project #07471)."
x26-ii) Outline informed consent procedures
"Participation will be anonymous and voluntary, and all subjects will previously provide written informed consent"
The informed consent form (in Portuguese) was attached as an Appendix.
X26-iii) Safety and security procedures

No significant risks for the participants.

X27-i) State the relation of the study team towards the system being evaluated

"InsuOnline© design and development was entirely founded by personal resources from the authors LAD, RMS, JBA, PAG, who are copyright holders. All authors contribute to design and evaluation of the game."
